# The mediating role of shame in the relationship between adolescent hairpulling and co‐occurring anxiety and depressive symptomology

**DOI:** 10.1002/jcv2.70041

**Published:** 2025-10-10

**Authors:** Talia F. Mayerson, Clare Mackay, Polly Waite

**Affiliations:** ^1^ Department of Experimental Psychology University of Oxford Oxford UK; ^2^ Department of Psychiatry University of Oxford Oxford UK

**Keywords:** adolescents, anxiety, depression, hairpulling, shame, trichotillomania

## Abstract

**Background:**

Hairpulling (HP) is a body‐focused repetitive behaviour that typically begins in early adolescence. However, most research on HP has focused on adults, with large gaps in our understanding of the phenomenology and mechanisms involved in HP in adolescence. A key empirically unexplained phenomenon in pediatric HP is the common co‐occurrence of depression, and anxiety symptoms. Shame is a prevalent self‐critical emotion in adults with BFRBs and a significant predictor of depression and anxiety in adolescence, and therefore may partially explain relationships between adolescent HP and depressive and anxiety symptoms.

**Methods:**

This cross‐sectional, survey‐based study examined HP phenomenology and the mediating role of shame in relationships between HP severity and symptoms of depression and anxiety in a community‐based sample of adolescents who hair‐pull. One hundred twenty‐eight adolescents aged 13–18 completed measures of HP phenomenology, HP severity, shame, depression symptoms, and anxiety symptoms.

**Results:**

Phenomenological findings demonstrated a relatively high prevalence of trance HP (61.8% usually/always), trichophagia (26.6% at least sometimes), and pulling from multiple sites (80.5%). Mediational analyses revealed HP severity was significantly associated with both depression (*β* = 0.355, *p* < 0.001) and anxiety symptoms (*β* = 0.266, *p* = 0.003). Shame was a significant partial mediator in the depression relationship (indirect effect = 0.192, 95% CI [0.078, 0.308]) and full mediator in the anxiety relationship (indirect effect = 0.146, 95% CI [0.053, 0.257]).

**Conclusions:**

Our phenomenological findings highlight the need for further examination of trance HP and other HP features in youth. Our mediational results suggest that shame may play a crucial role in relationships between HP and co‐occurring depression and anxiety symptomology in adolescence, underscoring the potential importance of targeting shame in improved HP interventions.

## Key Points

**What's Known**

- Despite being a critical age for the emergence of trichotillomania and co‐occurring symptoms, adolescents who hair‐pull have scarcely been studied in relation to hairpulling psychological aetiology and some phenomenological features.

**What's New**

- This study found that shame is a significant mediator in positive associations between hairpulling severity and depression and anxiety severity within a community sample of adolescents with clinical levels of hair‐pulling severity.
- It also found high levels of trance‐pulling and post‐pulling rituals.

**What's Relevant**

- Results align with cognitive‐behavioural models of HP, suggesting that negative self‐evaluation plays a crucial role in maintaining HP and co‐occurring symptoms.
- These findings suggest that therapeutic interventions which reduce shame and enhance one's ability to accept distressing internal experiences may be important in clinical approaches to adolescent hairpulling.

## Graphical abstract

This study found that shame is a significant mediator in positive associations between hairpulling severity and depression and anxiety severity within a community sample of adolescents with clinical levels of hair‐pulling severity. It also found high levels of trance‐pulling and post‐pulling rituals. Results align with cognitive‐behavioural models of HP, suggesting that negative self‐evaluation plays a crucial role in maintaining HP and co‐occurring symptoms.

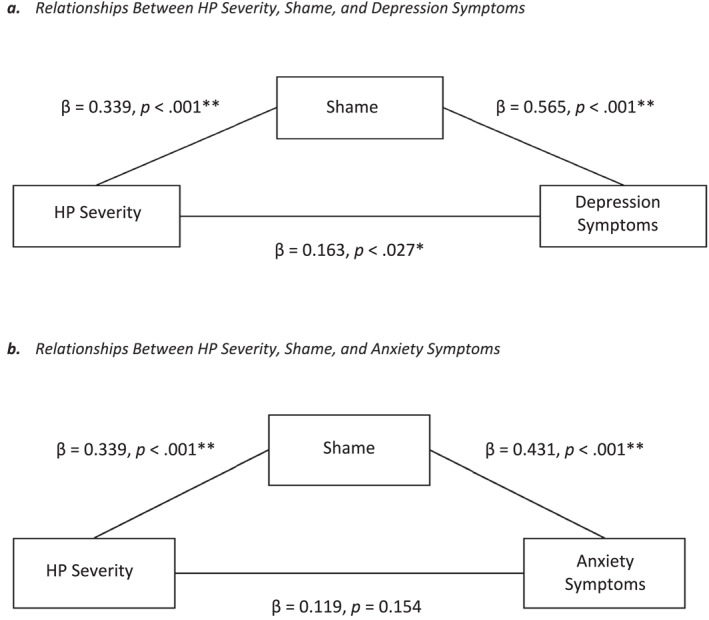

## INTRODUCTION

Hairpulling (HP) is an understudied body‐focused repetitive behaviour (BFRB) characterized by chronic and compulsive pulling of hair from one's body (Tolin, 2023). HP, diagnosed as trichotillomania at a clinical level, typically emerges in early adolescence, but most research exploring this BFRB has occurred in adults (American Psychological Association, 2013; Lin et al., 2023). One salient concern in paediatric HP is the common emergence and progression of co‐occurring mood and anxiety symptoms (Franklin et al., 2008; Panza et al., 2013). Co‐occurring depression and anxiety are frequent in young people with trichotillomania, estimated to occur in 16%–43% and 10%–32% of this population, respectively (Panza et al., 2013; Ricketts et al., 2022; Twohig et al., 2021). Significant associations have been observed between the severity of HP, depression, and anxiety symptoms starting in youth and extending into adulthood (Houghton et al., 2016; Ricketts et al., 2022; Schumer et al., 2015) when serious distress, psychosocial impairment, and functional interference is common (Woods, Flessner, et al., 2006).

It is currently unclear what psychological mechanisms facilitate the emergence of pathological relationships between HP and co‐occurring depressive and anxiety symptomology in youth. While no one theoretical model of HP is well‐established (Grant & Chamberlain, 2021), cognitive‐behavioural models have been steadily gaining empirical support and provide a useful framework for investigating the aetiology of these commonly co‐occurring symptoms (Crowe et al., 2024; Roberts et al., 2013; Sprich et al., 2023). Cognitive‐behavioural models suggest that HP is an operant behaviour maintained through a complex, cyclical interplay between dysfunctional HP cognitions, associated distress, negative self‐evaluation, and relief from distressing affective and/or physical internal states achieved through HP (Mansueto et al., 1997; Roberts et al., 2013). HP is conceptualised as a pathological self‐regulatory strategy: it temporarily abates discomfort while furthering eventual affective distress, namely harsh negative self‐evaluation (Crowe et al., 2024; Rehm et al., 2015, 2019).

Negative self‐evaluative emotions are integral in the manifestation of depression and anxiety symptoms in adolescence, and may therefore contribute to the emergence and maintenance of such co‐occurring symptomologies in adolescent HP (Gilbert & Irons, 2008; Muris & Meesters, 2014). In adults, shame has been observed as a particularly prevalent negative self‐evaluative emotion which is intricately related to HP throughout the pulling cycle (Bottesi et al., 2016; Houazene, 2021; Houazene et al., 2021; Noble, 2017; Weingarden & Renshaw, 2015). Shame arises from socially‐concerned, self‐critical cognitions (i.e., the perception of oneself as overall flawed, inadequate, or unworthy) (Gilbert & Irons, 2008), and experiences of shame can be classified into three subcategories: characterological (core self or identity), bodily (appearance and/or bodily functions) and behavioural (actions) (Andrews, 2002). Adults with BFRBs show high levels of all three shame types, and demonstrate heightened sensitivity to the induction of shame, increased likelihood for engaging in BFRBs after experiencing shame, and higher levels of shame after engaging in BFRBs than before (Bottesi et al., 2016; Houazene, 2021; Houazene et al., 2021; Noble, 2017). Significant correlations between shame and BFRB severity also suggest that more severe HP occurs with more intense experiences of shame (Noble, 2017; Singh, 2016).

In line with cognitive‐behavioural models, shame and HP may cyclically maintain each other in adolescence, a key period in the emergence of HP and co‐occurring depression and anxiety. Increased social concern, sensitivity to self‐consciousness, and harsh peer judgement are core experiences in adolescence and increase susceptibility to shame (Gilbert & Irons, 2008). Given that adults with BFRBs are already prone to heightened social concern, it is likely that adolescents who hair‐pull may experience shame with greater intensely (Mathew et al., 2021). Shame in adolescence is also significantly related to the development of depression and anxiety symptoms (P. Muris et al., 2015; Muris et al., 2018; Tilghman‐Osborne et al., 2008), and can predict depression over time (Nikolic et al., 2022). To date, shame has not been specifically examined in relation to HP nor co‐occurring symptoms in youth.

Beyond the dearth of research examining the aetiology of co‐occurring symptomology in youth HP, some core features of youth HP phenomenology are still largely unstudied. Common pulling sites in young people, for instance, have been identified (Franklin et al., 2008; Panza et al., 2013), but literature on post‐pulling behaviours is scarce, focussing mainly on trichophagia (Grant & Odlaug, 2008; Snorrason et al., 2021). While substantial work has been conducted to test a dichotomous model of HP ‘styles’ relating to levels of awareness while pulling (‘focused’/high awareness vs. ‘automatic’/low awareness pulling) (Flessner et al., 2007, 2008; Franklin et al., 2008; Lin et al., 2023; McGuire et al., 2020), trance pulling has yet to be studied as a potentially distinct phenomena. Pulling in a trance state is frequently reported in qualitative research and lived experience, described as a dissociative altered state of consciousness involving reduced awareness of the external world with varied, sometimes heightened awareness of internal functioning and behaviour (Rehm et al., 2015). Given that trance pulling is particularly challenging to control and may warrant unique therapeutic approaches, examining this pulling style with conceptual specificity in youth could provide clinically salient data.

In the present study, we aimed to (1) extend our understanding of the phenomenology of HP in adolescents, and (2) examine the role of shame as a mechanism for the relationship between HP and co‐occurring symptomology in a community sample of adolescents. We hypothesised that there would be a significant positive relationship between HP severity and depression symptom severity, and that this relationship would be significantly mediated by shame. Given the lack of theory or prior evidence to guide directional hypotheses, we also set out to explore whether shame mediated a relationship between HP and anxiety symptoms. We recruited an analogue population given low treatment‐seeking rates among people with HP and the dimensional nature of HP symptoms (Woods, Flessner, et al., 2006). As with obsessive‐compulsive symptoms (Abramowitz et al., 2014), community HP samples likely exhibit similar phenomenology to clinical samples, potentially providing data representative of broader HP populations.

## METHOD

### Design

We employed a cross‐sectional, survey‐based design to observationally investigate the mediating role of shame in the relationship between HP severity and symptoms of depression and anxiety in a community sample of adolescents who hair‐pull. Prior to beginning the study, we received approval (reference number: R91747/RE001) from the University of Oxford Medical Sciences Interdivisional Research Ethics Committee (MS IDREC). The study was pre‐registered on Open Science Framework and can be found publicly at https://doi.org/10.17605/OSF.IO/QW2KM. The pre‐registration did not include our first aim of exploring the phenomenology as the gaps in knowledge only became evident to us after the registration process.

### Lived experience involvement

Lived experience played a crucial role in this study. One researcher on the study team with trichotillomania contributed valuable insights from personal experience and community interactions, influencing the research aims, design, and interpretation of findings. An adolescent with hairpulling also provided input on the relevance of survey items and assisted with recruitment strategies. These perspectives were instrumental in shaping study development and execution.

### Participants

Participants were included if they were 13–18 years of age and self‐identified with experiencing HP that they found ‘difficult to control’. Given that our analyses benefit from a full range of dimensional data and that individuals with low severity pulling may still provide data on HP phenomenology, all participants who met our inclusion criteria were included in the final analysis regardless of their HP severity. No exclusion criteria were applied. Participant demographics are summarised in Table 1.

**TABLE 1:** Participant demographics.

	Total sample (*n* = 128)
Age (years)	
Mean (SD, range)	16.8 (1.3, 13–18)
Gender, *n* (%)	
Female	101 (78.9)
Male	14 (10.9)
Nonbinary	13 (10.2)
Preferred not to state	0 (0.0)
Ethnicity, *n* (%)	
Any White background	64 (50.0)
Any mixed background	32 (25.0)
Any Asian background	20 (15.6)
Any Black background	4 (3.1)
Any other background	3 (2.3)
Preferred not to state	7 (5.5)
Geographic region, *n* (%)	
United Kingdom	56 (43.8)
North America	43 (33.6)
Asia	11 (8.6)
Australia	8 (6.3)
Continental Europe	6 (4.7)
Other	2 (2.3)
Parental Socioeconomic Status, *n* (%)	
School completion (left school at age 15–16)	7 (5.5)
Further education (completed high school)	30 (23.4)
Higher education (completed university)	50 (39.1)
Post‐graduate qualification	31 (24.2)
Unknown	10 (7.8)

*Note*: Geographic regions are based on participants' self‐reported current residence. Socioeconomic status is approximated using parent/guardian's educational attainment as a proxy measure.

### Recruitment

Recruitment occurred from May‐June 2024 via online advertisements posted on social media, BFRB forums, and relevant charity websites (see Supporting Information S1: Table S1). The advertisements brought prospective participants to a Qualtrics landing page where they were stratified by age for the consent process. Participants aged 16–18 provided informed consent as they were deemed competent youths. Participants aged 13–15 provided informed assent while parents or guardians provided informed consent on their behalf.

### Procedure

After consent/assent, participants could opt into a prize draw for a chance to win one of four £20 Amazon voucher. They then provided demographic information and went on to complete the core survey measures. Post‐survey, participants received resources for HP and general adolescent mental health.

### Measures

#### Hairpulling phenomenology questionnaire

The Hairpulling phenomenology questionnaire (HPQ) is an original measure created in collaboration with our lived experience consultants and used to descriptively characterise adolescent HP phenomenology in our sample (See Supporting Information S1: Table S2). We derived this 5‐item instrument through construction of novel items for assessing HP features without clear previous examination, and synthesis and modification of phenomenological items utilised in previous HP phenomenological work in youth (Franklin et al., 2008). The first two items are rated on a three‐point (0–2) Likert scale and provide descriptive insight into HP distress and functional interference, particularly in relation to shame and trance pulling. While similar to items used in previous works, these items were modified to resonate more directly with individuals' lived experience. The remaining three items are rated on a 4‐point scale (0–3) and assess various aspects of HP phenomenology, including the identification of pulling sites using a list of options derived in Franklin et al. (2008) and novel items regarding pulling styles and behaviours. While the HPQ has not been psychometrically validated, it provides valuable descriptive data on HP aspects not covered by established HP measures.

#### Trichotillomania scale for children—child report

We employed the Trichotillomania scale for children—child report (TSC‐C) for measuring overall HP severity (Tolin et al., 2008). This instrument consists of 12 self‐report items, each scored on a 3‐point (0–2) Linkert scale. The TSC‐C yields two sub scores (HP severity and HP distress/impairment) that are averaged separately and then summed to create a total HP severity score ranging from 0 to 4. For our mediation analyses, the total TSC‐C score was used as a measure of overall HP severity. When tested in samples of youth with non‐clinical and clinical HP, the TSC‐C has demonstrated good internal consistency (clinical: *a* = 0.82, non‐clinical: *a* = 0.83), test re‐test reliability (*r* = 0.89 [clinical only]), and good convergent validity with other measures of HP (Tolin et al., 2008). In our sample, internal consistency of the TSC‐C was good (*a* = 0.82). While no singular self‐report measure of HP is considered well‐established in young people, the TSC‐C is one of the most widely used and psychometrically sound instruments available for assessing HP severity in adolescents (McGuire et al., 2012).

#### Experiences of shame scale

Shame was measured using a shortened 12‐item version of the Experiences of Shame Scale (ESS) (Andrews, 2002; Nikolic et al., 2022). The ESS produces a total shame score and three sub scores: characterological, behavioural, and bodily shame (Andrews, 2002). For our mediation analyses, the total ESS score was used. Each item is scored on a four‐point (1–4) Likert scale, with total scores ranging on from 12—48. ESS items are phrased as statements regarding various experiences of shame as they have occurred over the past year (e.g., ‘I try to cover‐up or conceal some of my personal habits’). In samples of adults who hair‐pull, the ESS has been the primary measure used (Houazene, 2021; Houazene et al., 2021; Noble, 2017; Singh, 2016). In its original 25‐item form, the ESS has demonstrated excellent internal consistency (*α* = 0.92), and good test‐retest reliability (*r* = 0.83) and construct validity in adults (*n* = 162) (Andrews, 2002). In youth, psychometric properties of the ESS have not been assessed in full. However, one study using the shortened 12‐item version found good internal consistency (*α* = 0.86) in adolescents (Nikolic et al., 2022). In our sample, internal consistency was excellent (*α* = 0.91).

#### Short mood and feelings questionnaire

We assessed depression symptoms with the Short Mood and Feelings Questionnaire (SMFQ) (Angold, 1995). This 13‐item self‐report measure is a well‐validated shortened version of the Moods and Feelings Questionnaire, currently recommended in the UK by the National Institute for Health and Clinical Excellence as a screening tool for depression in youth (Hopkins et al., 2015). The SMFQ consists of statement items (e.g., ‘I felt miserable or unhappy’) rated on a three‐point scale (0 = not true, 1 = sometimes, 2 = true) (Angold, 1995). Total scores range from 0 to 26, though a score of 8 or above is typically used as a cut‐off to identify clinically significant symptoms (Angold, 1995). In community samples of young people, the SMFQ has demonstrated strong psychometric properties, such as good internal consistency (*α* = 0.88), strong construct validity through factor analysis, and high criterion validity with clinical depression diagnoses (Turner et al., 2014). In our sample, internal consistency was also good (*α* = 0.89).

#### Generalised Anxiety Disorder—7 items

We measured anxiety symptoms with the generalised anxiety disorder—7 items (GAD‐7), a 7‐item self‐report instrument which assesses the frequency of anxiety symptoms over the past 2 weeks (Spitzer, 2006). It consists of items (e.g., ‘Feeling nervous, anxious, or on edge’) rated on a four‐point scale (0 = not at all, 1 = several days, 2 = more than half the days, 3 = nearly every day) (Spitzer, 2006). Total scores range from 0 to 21, with a score of 11 typically used as a cut‐off for identifying moderate anxiety symptoms and likelihood for a diagnosis in young people (Mossman et al., 2017). In community samples of youth and young adults, the GAD‐7 has demonstrated good psychometric properties, including excellent internal consistency (*α* = 0.922), unidimensional structure, and good criterion validity (aged 15–24) (Ip et al., 2022). In our sample, internal consistency was excellent (*α* = 0.90).

### Data analysis

All analyses were conducted in R studio version 4.3.1. Data were initially included if at least 80% of items on each core variable measure were completed. Remaining data cases were then screened for appropriateness using current guidelines for identifying imposter participants, and suspicious cases were deleted listwise (see Supporting Information S1: Figure S1) (Ridge et al., 2023). We identified suspicious cases based on whether they exhibited patterns identified by Ridge et al. (2023). Common reasons for exclusion included identically configured email addresses, response bursts with similar responses immediately following an advertisement post, straight‐lining, and failed bot detection items or other non‐sensible responses (e.g., identifying that they found the study through a recruitment route not used). There were no (0.0%) missing data on core variables of the participant cases retained for analysis. To examine phenomenology, we calculated means and standard deviations. Meditation analyses were conducted using the PROCESS macro protocol, and analyses for depression and anxiety symptoms were run separately (Hayes, 2021; R Core Team, 2023). The indirect (mediation) effect was estimated using 5000 bootstrap samples, and bias‐corrected 95% confidence intervals were used to assess its significance (Hayes, 2021). This approach is robust to violations of the data normality assumption common in mediational datasets (Hayes, 2021). PROCESS macro uses ordinary least squares (OLS) regression to estimate model coefficients, thus we tested OLS assumptions for each regression model relevant to our aims (Hayes, 2021; R Core Team, 2023).

## RESULTS

Descriptive HP characterization statistics are provided in Table 2. Adolescents most commonly pulled from their scalp (75.0%, 95% CI [67.5, 82.5]), followed by the pubic area (57.0%, 95% CI [48.4, 65.6]), eyebrows (46.9%, 95% CI [38.2, 55.6]), and eyelashes (46.1%, 95% CI [37.5, 54.7]). Less frequent pulling sites included legs (34.4%, 95% CI [26.2, 42.6]), armpits (25.8%, 95% CI [18.2, 33.4]), arms (18.8%, 95% CI [12.0, 25.6]), trunk (10.9%, 95% CI [5.5, 16.3]), beard and other unspecified areas (both 7.0%, 95% CI [2.6, 11.4]), and moustache (3.1%, 95% CI [0.1, 6.1]). Most participants (80.5%, 95% CI [73.6, 87.4]) reported pulling from multiple sites (see Supporting Information S1: Table S3). Regarding pulling styles, half the participants reported ‘usually’ or ‘always’ pulling without realising they are doing it, and over three quarters reported usually or always searching out hairs with a particular feeling to pull. Nearly two thirds reported at least usually finding themselves in a trance when pulling. Post‐pulling rituals were common, with over three quarters of participants at least usually looking at the hair/root and nearly two thirds usually or often rubbing the hair or root afterwards. Although less common, just under half the partipants had bitten the hair or root or put it in their mouth and just over a quarter had eaten the hair or root.

**TABLE 2:** Hairpulling awareness, post‐pulling rituals, shame and functional interference from responses on the hairpulling phenomenology questionnaire (*n* = 128).

Pulling awareness	*M* (SD)	*N* (%)			
		Never	Sometimes	Usually	Always
I typically pull without realising that I'm doing it.	1.5 (0.8)	11 (8.6)	53 (41.4)	48 (37.5)	16 (12.5)
I search out hairs that have a particular feeling to pull out.	2.3 (0.9)	6 (4.7)	57 (44.5)	40 (31.3)	67 (52.3)
I find myself in a sort of ‘trance’ when pulling, where I am unaware of the world around me.	1.8 (1.0)	12 (9.4)	37 (28.9)	39 (30.5)	40 (31.3)
Post‐pulling rituals					
I look at the hair or hair root.	2.2 (1.0)	9 (7.0)	20 (15.6)	34 (26.6)	65 (50.8)
I rub the hair or hair root against my fingertips, face, or lips.	1.8 (1.1)	22 (17.2)	29 (22.7)	29 (22.7)	48 (37.5)
I bite the hair (or hair root) or put it in my mouth.	0.9 (1.2)	73 (57.0)	22 (17.2)	11 (8.6)	22 (17.2)
I eat the hair or hair root	0.6 (1.0)	94 (73.4)	10 (7.8)	12 (9.4)	12 (9.4)
HP shame					
How easy do you find it to talk openly about your hairpulling?	1.28 (0.76)	–	–	–	–
HP Functional Interference					
Did you find yourself being late or missing an activity because you were ‘stuck’ in a pulling episode?	0.76 (0.75)	–	–	–	–

*Note*: Pulling awareness and post‐pulling ritual items were rated on a 4‐point Likert scale (0–3), from ‘never’ to ‘always’. HP shame and functional interference items were rated on a 3‐point Likert scale (0–2).

Descriptive statistics for all core measures can be found in Table 3. Pearson correlations between variables are provided in Table 4, with all variables significantly correlated in the expected directions.

**TABLE 3:** Descriptive statistics for mediational variables.

Measure	Score type	Full sample (*n* = 128)		
		*M*	SD	Observed range
HP severity (TSC‐C)	Total	2.5	0.7	0–3.9
	Severity	1.5	0.4	0–2
	Distress/Impairment	1.0	0.5	0–2
Shame (ESS)	Total	36.0	8.0	12–48
	Characterological	11.6	3.2	4–16
	Bodily	11.4	3.1	4–16
	Behavioural	11.6	3.2	4–16
Depression symptoms (SMFQ)	Total	14.1	6.5	0–26
Anxiety symptoms (GAD‐7)	Total	11.9	5.3	0–21

*Note*: ESS = Experiences of Shame Scale; GAD‐7 = Generalised Anxiety Disorder‐7; SMFQ = Short Mood and Feelings Questionnaire; TSC‐C = Trichotillomania Scale for Children—Child Report.

**TABLE 4:** Pearson correlation matrix between mediational variables (*n* = 128).

Variable	1	2	3	4
1. HP severity	1	‐	‐	‐
2. Shame	0.339**	1	‐	‐
3. Depression symptoms	0.355**	0.620**	1	‐
4. Anxiety symptoms	0.266**	0.472**	0.637**	1

*Note*: HP severity was measured using the TSC‐C total score. Shame was measured using the ESS total score. Depression symptoms were measured with the SMFQ total score. Anxiety symptoms were measured using the GAD‐7 total score. **Correlation is significant at the 0.001 level.

### Shame as a mediator between HP and depression symptoms

**FIGURE 1:**
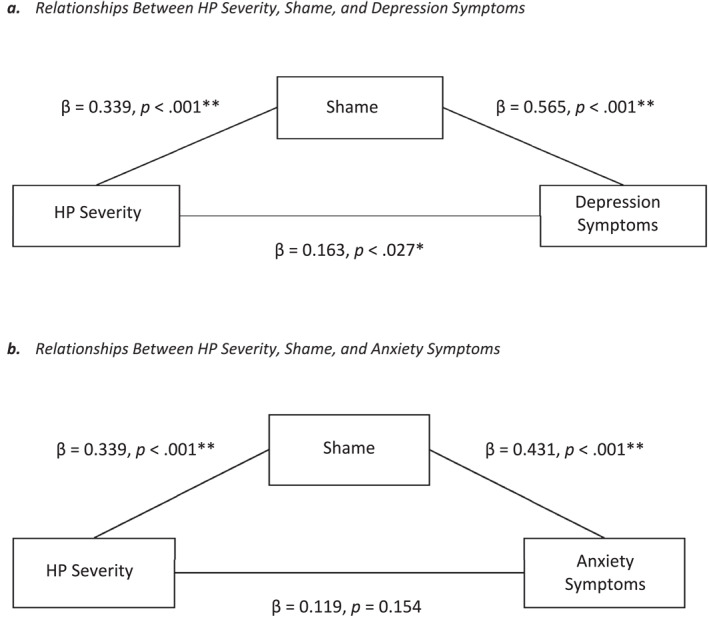
(A) Relationships Between HP Severity, Shame, and Depression Symptoms. (B) Relationships Between HP Severity, Shame, and Anxiety Symptoms. All path coefficients are standardised. *Significance is at *p* < 0.05; **Significance is at *p* < 0.001. HP severity was measured using the TSC‐C total score. Shame was measured with the experiences of shame scale total score. Depression symptoms were assessed with the short mood and feelings questionnaire total score. Anxiety symptoms were measured with the GAD‐7 total score.

Relationships between TSC‐C total score (HP severity), ESS total score (experiences of shame), and SMFQ total score (depression symptoms) are depicted in Figure 1A. Regression and mediation statistics are presented in Table 5.

**TABLE 5:** Regression and mediational results of HP severity (TSC‐C), shame (ESS), and depression symptoms (SMFQ) mediation model (*n* = 128).

Regression analysis	*β*	SE	*t*	*p*	95% confidence interval	
					Lower	Upper
HP on shame	0.339	0.084	4.046	<0.001	0.173	0.505
Shame on depression	0.565	0.059	7.727	<0.001	0.420	0.710
HP on depression	0.163	0.073	2.233	0.027	0.019	0.308

Mediation analysis	*β*	SE	*t*	*p*	95% confidence interval	
					Lower	Upper
Total effect of HP on depression	0.355	0.764	4.261	<0.001	0.190	0.520
Direct effect of HP on depression	0.163	0.073	2.233	0.027	0.019	0.308

Indirect effects of HP on depression	Effect	Bootstrapped SE	Bootstrapped 95% confidence interval	
			Lower	Upper
Via shame	0.192	0.058	0.078	0.308

*Note*: All reported coefficients are standardised. HP severity was measured using the TSC‐C total score. Shame was measured with the ESS total score. Depression symptoms were assessed with the SMFQ total score.

As hypothesised, there was a significant positive relationship between HP severity and depression symptoms (*β* = 0.355, *p* < 0.001) and HP severity was also positively associated with shame (*β* = 0.339, SE = 0.084, *p* < 0.001, 95% CI [0.173, 0.505]). The indirect effect of HP severity on depression symptoms through shame was significant (standardised indirect effect = 0.192, bootstrapped SE = 0.058, bootstrapped 95% CI [0.078, 0.308]). The direct effect of HP severity on depression symptoms remained significant after accounting for the mediating role of shame (*β* = 0.163, SE = 0.0732, *p* = 0.027, 95% CI [0.019, 0.308]), which indicated that shame partially mediated the relationship between HP severity and depression.

### Shame as a mediator between HP and anxiety symptoms

The relationships between TSC‐C total score (HP severity), ESS total score (experiences of shame), and GAD‐7 total score (anxiety symptoms) are depicted in Figure 1B. Regression and mediation statistics are presented in Table 6.

**TABLE 6:** Regression and mediational results of HP severity (TSC‐C), shame (ESS), and anxiety symptoms (GAD‐7) mediation model (*n* = 128).

Regression analysis	*β*	SE	*t*	*p*	95% confidence interval	
					Lower	Upper
HP on shame	0.339	0.084	4.046	<0.001	0.173	0.505
Shame on anxiety	0.431	0.084	5.186	<0.001	0.267	0.596
HP on anxiety	0.119	0.083	1.435	0.154	−0.045	0.284

Mediation analysis	*β*	SE	*t*	*p*	95% confidence interval	
					Lower	Upper
Total effect of HP on anxiety	0.266	0.086	3.092	0.003	0.096	0.435
Direct effect of HP on anxiety	0.119	0.083	1.435	0.154	−0.045	0.284

Indirect effects of HP on anxiety	Effect	Bootstrapped SE	Bootstrapped 95% confidence interval	
			Lower	Upper
Via shame	0.146	0.053	0.053	0.257

*Note*: All reported coefficients are standardised. HP severity was measured using the TSC‐C total score. Shame was measured with the ESS total score. Depression symptoms were assessed with the SMFQ total score.

There was a significant positive relationship between HP severity and anxiety symptoms (*β* = 0.266, *p* = 0.003). The indirect effect of HP severity on anxiety symptoms through shame was significant (standardised indirect effect = 0.146, bootstrapped SE = 0.053, bootstrapped 95% CI [0.053, 0.257]). The direct effect of HP severity on anxiety symptoms was nonsignificant after accounting for the mediating role of shame (*β* = 0.119, SE = 0.083, *p* = 0.154, 95% CI [‐0.045, 0.284]), indicating that shame fully mediated the relationship between HP and anxiety.

## DISCUSSION

In this study, we examined HP phenomenology and assessed cross‐sectional relationships between HP severity, shame, and symptoms of depression and anxiety in an internet‐based community sample of adolescents who experience HP. We extended the phenomenological understanding of HP in terms of common pulling‐sites, pulling styles, and post‐pulling rituals. We found a significant positive association between HP severity and depression symptoms which was partially mediated by shame. We also found a significant positive association between HP severity and anxiety symptoms, and this relationship was fully mediated by shame.

Participants in this study reported frequent pulling sites (i.e., scalp, brows, lashes) in line with a similarly sized Internet sample of youth who hair‐pull at a proxy‐clinical level and a small treatment‐seeking clinical sample of young people (Franklin et al., 2008; Panza et al., 2013). Pulling from the pubic area was more frequent in our sample, possibly related to a higher proportion of older youth than previous work or to a cohort effect. Interestingly, we also found that pulling from multiple sites was common, occurring in over 80% of the sample. This may also be explained by our use of older adolescents, as the number of pulling sites has been observed to increase in relation to age (Panza et al., 2013). We also found post‐pulling rituals to be frequent, with trichophagia occurring at least some of the time in over a quarter of participants, consistent with rates reported in older adolescents and adults with trichotillomania (Grant & Odlaug, 2008).

To our knowledge, this is the first study examining trance pulling with conceptual specificity in youth and we found this to be extremely common. Most (88%) participants reported pulling in a trance at least some of the time. This phenomenon may be pertinent to understanding shame manifestation in HP. For instance, experimental and observational data suggests that shame is a trigger for trance‐like, dissociative states in other populations who have difficulty with self‐regulation (e.g., post‐traumatic stress disorder), as it is a particularly difficult emotion to regulate (Kouri et al., 2023; Rudy et al., 2022). It is possible that shame serves as a powerful pulling trigger for adolescents who pull in a trance. Further, it's interesting that over half (61.8%) of the sample reported pulling in a trance ‘usually’ or ‘always’ while less (50%) participants indicated ‘usually’ or ‘always’ pulling without realising. This may suggest a qualitative distinction between the experience of pulling in a trance and pulling with low awareness. More comprehensive examinations of this phenomenological feature in relation to shame and its distinct qualities from other pulling styles are accordingly well‐motivated.

We found for the first time that HP is significantly related to shame in adolescents. To our knowledge, an examination of shame in adolescents with any type of BFRB has not been conducted, though several such studies have been caried out in adults (Houazene, 2021; Houazene et al., 2021; Noble, 2017). We found bodily, behavioural, and characterological shame reached a similar level on average, suggesting that all contribute to adolescent HP with equal strength. In our analyses, HP severity showed a moderately sized (*r* = 0.34) significant association with total shame scores, suggesting that higher levels of shame were associated with more severe HP. In cross‐sectional shame studies in adults with clinical BFRBs, associations between BFRB severity and shame have been small to moderate in size (Houazene, 2021; Houazene et al., 2021; Noble, 2017). While it may be merely a reflection of differences in the methodology, our medium sized effect marginally exceeds that of findings with adults and could support the hypothesis that HP‐related shame manifests with greater intensity in adolescence.

Unsurprisingly, HP severity was significantly associated with symptoms of both depression and anxiety in our sample, which is consistent with other studies using clinical and proxy‐clinical samples of youth who hair‐pull (Houghton et al., 2016; Ricketts et al., 2022; Schumer et al., 2015; Woods, Flessner, et al., 2006). In our sample, individuals reporting more severe HP also reported more symptoms of depression and anxiety, often exhibiting SMFQ and GAD‐7 scores above the cut‐offs for predicted clinical significance (Angold, 1995; Mossman et al., 2017; Spitzer, 2006). Further, while the TSC‐C does not have a particular clinical threshold score, it is notable that average overall severity scores in our sample (*M* = 2.5) actually exceeded those (*M* = 2.11) in a smaller clinical sample of youth (Tolin et al., 2008).

As hypothesised, we found that shame significantly mediates associations between HP severity and co‐occurring depression and anxiety symptoms in adolescence at one point in time. Shame may facilitate more severe depression and anxiety symptoms in adolescent HP through pathological processes of internalisation (i.e., inward‐facing cognitions such as ‘I'm stupid’, ‘I should be able to control myself’, ‘other people will notice and reject me’). Rumination, negative cognitive distortions, and social isolation and avoidance are elicited by shame and commonly experienced in depression and anxiety disorders (Joubert et al., 2022; Muris & Meesters, 2014; Orth et al., 2006). Shame‐facilitated social isolation and avoidance raise particular concern in HP as it is evident that HP‐related shame commonly interferes with social engagement throughout the lifetime (Casati et al., 2000; Li et al., 2023; Roohafza et al., 2014; Stemberger et al., 2000; Tolin, 2023).

Importantly, the impact of stigma in HP may amplify shame and thus contribute to the development of co‐occurring mood and anxiety symptoms. Stigmatisation is significantly associated with worsened depression and anxiety symptoms in the general population, with some data suggesting it serves as a maintenance factor for these disorders (Alonso et al., 2008; Dolezal, 2022). It is salient that HP remains a societally stigmatised condition across cultural contexts (Ricketts et al., 2012). As has been recommended by clinicians and patients alike, increasing societal awareness and understanding of BFRBs is an important step to reducing shame and stigma (Mackay, 2023).

### Strengths and limitations

This study benefitted from several strengths. The targeted yet inclusive recruitment strategy ensured the sample included a wide range of young people who on average experienced HP at similar or higher levels of severity to clinical and proxy‐clinical samples (Tolin et al., 2008), and were more diverse in gender, ethnic identity, and geographic residence than in any other youth HP study to our knowledge. Implementation of a robust data screening process alongside the internet‐mediated design reduced the likelihood of incorrectly including imposter participants (Ridge et al., 2023). Substantial involvement of people with lived HP experience enhanced the practical relevance of our research aims, original instrument, and survey.

Simultaneously, this research faced limitations. Our remote, semi‐anonymous recruitment rendered verification of participant identities challenging, leading us to exclude many participants and constraining our sample size. Our recruitment strategy also relied heavily on participants from social media, potentially biassing our sample to represent the experiences of specific online communities rather than the broader population of adolescents with HP. Despite improved diversity, our sample remained skewed towards White, middle‐class females from Western nations, precluding us from exploring gender and/or ethnic/cultural effects. While this gender ratio (80% female) aligns with some recent youth HP research, it remains unclear whether this reflects a true gender distribution in the community or sampling bias (Ricketts et al., 2023). Furthermore, all constructs in this work were measured via self‐report which introduces the threat of common method variance and could have inflated correlations between our key variables. Similarly, to integrate lived experience, we consulted only two informants whose experiences are not necessarily representative of all people who hair‐pull. Finally, our cross‐sectional design precluded causal inferences regarding the observed variable relationships as chronology of symptom manifestation and symptom changes over time could not be assessed. Accordingly, our data cannot be used to comprehensively assess the aetiology of HP with co‐occurring mood and anxiety symptoms in youth.

### Implications

As an understudied and underdiagnosed condition with no well‐established interventions, research on HP that builds a comprehensive theoretical model in youth will inform the development of effective interventions (Grant & Chamberlain, 2021). While our research provides evidence that shame may underpin relationships between HP and some co‐occurring symptoms in adolescence within a cognitive‐behavioural framework, there is more to do. In particular, longitudinal research assessing HP, and co‐occurring symptoms throughout adolescence and early adulthood is required to examine the observed developmental progression of this symptomology and help establish a more robust HP model incorporating negative self‐evaluation (e.g., shame) as a mechanism for mood and anxiety symptom emergence. Furthermore, while shame has demonstrated a closer relationship with depressive and anxiety symptoms than other self‐critical emotions (e.g., guilt) in youth, these emotions can demonstrate varying levels of convergence in young people (Peter Muris et al., 2015; Tilghman‐Osborne et al., 2008). Future research should measure a range of negative self‐evaluative constructs in relation to HP and co‐occurring symptoms to identify other potentially significant mediators.

If shame both maintains HP severity directly and contributes to the emergence of co‐occurring symptoms, this emotion and its regulation would be a critical target for treatment. Habit reversal therapy (HRT) is currently considered by experts as the gold‐standard treatment for BFRBs, but has rarely been shown to simultaneously eradicate co‐occurring symptomology, and its longitudinal efficacy is unclear (Farhat et al., 2020; Grant & Chamberlain, 2021; Usmani et al., 2023). HRT is designed to focus attention on unwanted behaviour and its consequences, as well as lack of control. For many individuals, this process may be both ineffective and inadvertently cause more shame, particularly in patients whose pulling style may already include some level of awareness with limited control (e.g., those who pull in a trance). Recent studies suggest that acceptance commitment therapy (ACT), which instead aims to directly improve emotional self‐regulatory strategies through acceptance of distressing internal experiences (Usmani et al., 2023), is effective in adults with HP (Lee et al., 2020; Twohig et al., 2021; Woods, Wetterneck, & Flessner, 2006). Research establishing whether the reduction of shame moderates the efficacy of cognitive‐behavioural psychotherapies in young people is warranted.

### Conclusion

This study provides novel characterization of HP phenomenology and evidence for the potentially salient role that shame may play in maintaining HP and co‐occurring symptomology in adolescence. We found that HP severity was significantly associated with experiences of shame, and that shame in turn significantly predicted both depression and anxiety symptoms. Our mediational analyses confirmed that shame partially mediated the relationship between HP severity and depression symptoms and fully mediated the relationship between HP severity and anxiety symptoms. While future longitudinal research is needed to examine relationships between shame, HP, and co‐occurring symptoms in youth over time, our results contribute to an empirical basis from which such studies could be executed. Ultimately, working toward a nuanced understanding of shame in HP across diverse populations may lead to more effective interventions for this highly stigmatised, psychosocially deleterious condition.

## AUTHOR CONTRIBUTIONS

**Talia F. Mayerson**: Conceptualization; data curation; formal analysis; investigation; methodology; project administration; resources; visualization; writing—original draft. **Clare Mackay**: Conceptualization; methodology; supervision; writing—review and editing. **Polly Waite**: Conceptualization; methodology; supervision; visualization; writing—review and editing.

## CONFLICT OF INTEREST STATEMENT

The authors declare no conflicts of interest.

## ETHICAL CONSIDERATIONS

Ethical approval was provided for this research by the University of Oxford Medical Sciences Division Ethics Committee (R91747) in April 2024. Informed consent was obtained for all participants aged 16–18. For participants aged 13–15, informed assent was obtained from participants and informed consent was obtained from their parent or guardian.

## Supporting information

Supporting Information S1

## Data Availability

The data that support the findings in this research will be available on the Open Science Framework by 2026, following further longitudinal research with the study sample.
