# Supplementary material for: The mediating role of shame in the relationship between adolescent hairpulling and co‐occurring anxiety and depressive symptomology
Source: JCPP Adv. 2025 Oct 10;6(2):e70041. doi: 10.1002/jcv2.70041 (PMC13260712; doi:10.1002/jcv2.70041)
Supplement: Supplementary file 1 — Supporting Information S1 [file JCV2-6-e70041-s001.docx]

The Mediating Role of Shame in the Relationship between Adolescent Hairpulling and Co-occurring Anxiety and Depressive Symptomology

Talia Mayerson, Clare Mackay, Polly Waite

**Electronic Supporting Information**

Figure S1

*Flow Diagram of Participant Responses by Data Screening Stage*

***
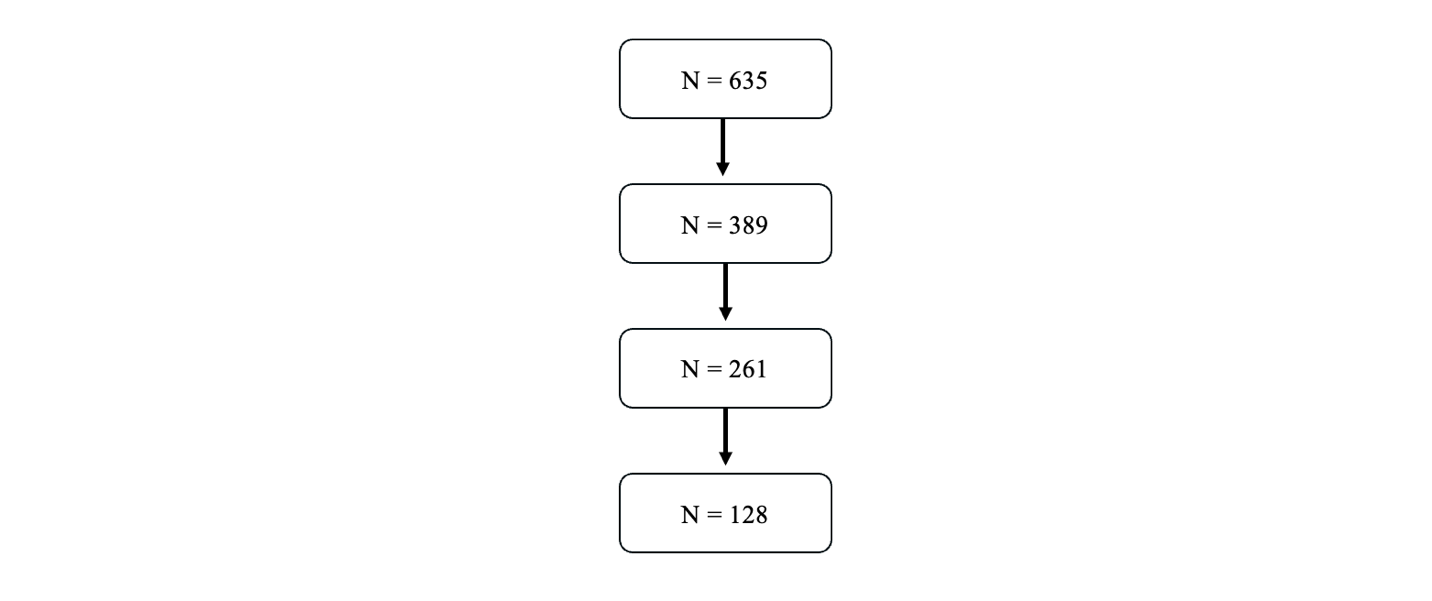
***

Initial responses

Complete responses

Responses removed for suspicious characteristics

N = 635

N = 389

N = 261

N = 128

Responses retained for final analysis

**Stage 2**

**Stage 4**

**Stage 3**

**Stage 1**

# *Note. “*Complete responses” were data cases with less than 20% data missing from core variables. Suspicious data included cases where maximum or minimum items were chosen consistently across measures, email formulation was homogenous across multiple entries in proximity, and/or bot detection items were failed (e.g., a 0.5 or lower response on the Qualtrics built-in reCAPTCHA).

**Table S1**

*Participant Responses by Recruitment Route and Data Screening Stage*

| Recruitment Route | Complete responses  *(n* = 389) | | Retained responses  (*n* = 128) | |
| --- | --- | --- | --- | --- |
|  | **N** | **%** | **N** | **%** |
| Social media platforms | 315 | 81.0 | 118 | 92.2 |
| Instagram | 73 | 18.8 | 41 | 32.0 |
| Facebook | 107 | 27.5 | 6 | 4.7 |
| Reddit | 107 | 27.5 | 68 | 53.1 |
| Discord | 19 | 4.9 | 1 | 0.8 |
| X | 9 | 2.3 | 2 | 1.6 |
| Charity mailing lists or websites | 46 | 11.8 | 2 | 1.6 |
| Referral between participants | 8 | 2.1 | 4 | 3.1 |
| Unknown | 20 | 5.1 | 5 | 3.9 |

***Note.*** “Complete responses” refer to data cases retained based on adequate completion (i.e., completion of at least 80% of items on all core variable measures). “Retained Responses” refers to data cases retained for data analysis. Incomplete data cases did not have data on the recruitment route by which the participant found the survey. Recruitment via Instagram primarily took place via trichotillomania influencers’ posts and stories, while Reddit recruitment occurred through paid advertisements and repeated posts in BFRB-related sub-reddit forums (e.g., “r/trichotillomania,” “r/trichsters,” “r/calmhands,” etc.). While it may appear that many of the participants found through Facebook and charities were not retained for analysis, it is important to note that participants self-reported the recruitment route by which they found the survey. Thus, bots and other inauthentic participants may have chosen “Facebook” or “Charity” at random despite having found the survey via another site or platform.

**Table S2**

*Hairpulling Phenomenology Questionnaire (HPQ)*

| ***HPQ Item*** | **Response Options** |
| --- | --- |
| 1. How easy do you find it to talk openly about your hair-pulling? | (0) I find it easy to talk about my hairpulling with people in my life.  (1) Sometimes I find it difficult to talk about my hairpulling with people in my life.  (2) I find it impossible to talk about my hairpulling with people in my life. |
| 2. Did you find yourself being late or missing an activity because you were ‘stuck’ in a pulling episode? | (0) I was never late or missed an activity because I was ‘stuck’ in a pulling episode.  (1) I was almost late or nearly missed an activity because I was ‘stuck’ in a pulling episode.  (2) I was late or missed activity one or more times because I was ‘stuck’ in a pulling episode. |
| 3. How do you typically pull-out hair? Please rate how often you find yourself hair-pulling like the examples below with the option that best describes your typical hair-pulling. | |
| I typically pull without realizing that I’m doing it. | (0) never  (1) sometimes  (2) usually  (3) all of the time |
| I search out hairs that have a particular feeling to pull out. | (0) never  (1) sometimes  (2) usually  (3) all of the time |
| I find myself in a sort of ‘trance’ when pulling, where I am unaware of the world around me and/or time passing. | (0) never  (1) sometime es  (2) usually  (3) all of the time |
| 4. After you pull out a hair, what do you do? Please rate how often you find yourself doing the following after you pull out a hair. | |
| I look at the hair or hair root. | (0) never  (1) sometimes  (2) usually  (3) all of the time |
| I rub the hair or hair root against my fingertips, face, or lips. | (0) never  (1) sometimes  (2) usually  (3) all of the time |
| I bite the hair (or hair root) or put it in my mouth. | (0) never  (1) sometimes  (2) usually  (3) all of the time |
| I eat the hair or hair root. | (0) never  (1) sometimes  (2) usually  (3) all of the time |
| 5. Where do you typically pull hair from? You may choose as many options as you like. | Scalp, Brows, Lashes, Pubic, Legs, Arms, Armpits, Trunk, Moustache, Beard, Other |

***Note.*** Instructions were as follows: “We’d like to ask you a few more questions about your hair-pulling. The following items have been designed specifically for this study to explore some aspects of hairpulling that often are not addressed by other existing questionnaires. Please read each question carefully and choose the statement that you think best describes you.”

**Table S3**

*Number of Bodily Sites from Which Hair is Pulled*

| Number of Pulling Sites | Full Sample (*n* = 128) | |
| --- | --- | --- |
|  | **N** | **%** |
| 1 | 25 | 19.5 |
| 2+ | 103 | 80.5 |
| 3+ | 84 | 65.6 |
| 4+ | 56 | 43.8 |
| 5+ | 29 | 22.7 |
| 6+ | 17 | 13.3 |
| 7+ | 6 | 4.7 |
| 8+ | 4 | 3.1 |
| 9+ | 3 | 2.3 |
| 10+ | 3 | 2.3 |
| 11+ | 0 | 0.0 |

***Note.*** N = frequency count. Percentages in each column sum to more than 100% because categories are not mutually exclusive (e.g., someone with 3 pulling sites is counted in both the “2+” and “3+” rows).
